# Pregnancy in obese women and mechanisms of increased cardiovascular risk in offspring

**DOI:** 10.1093/eurheartj/ehae671

**Published:** 2024-11-07

**Authors:** Anna L K Cochrane, Michael P Murphy, Susan E Ozanne, Dino A Giussani

**Affiliations:** Department of Physiology, Development and Neuroscience, University of Cambridge, Downing Street, Cambridge CB2 3EG, UK; Department of Medicine, University of Cambridge, Hills Road, Cambridge CB2 0QQ, UK; Department of Medicine, University of Cambridge, Hills Road, Cambridge CB2 0QQ, UK; MRC Mitochondrial Biology Unit, University of Cambridge, Hills Road, Cambridge CB2 0XY, UK; Metabolic Research Laboratories and MRC Metabolic Diseases Unit, Institute of Metabolic Science, University of Cambridge, Cambridge, UK; Loke Centre for Trophoblast Research, University of Cambridge, Downing Street, Cambridge CB2 3EG, UK; Cambridge Strategic Research Initiative in Reproduction, University of Cambridge, Cambridge, UK; British Heart Foundation, Cambridge Cardiovascular Centre for Research Excellence, University of Cambridge, Cambridge, UK; Department of Physiology, Development and Neuroscience, University of Cambridge, Downing Street, Cambridge CB2 3EG, UK; Metabolic Research Laboratories and MRC Metabolic Diseases Unit, Institute of Metabolic Science, University of Cambridge, Cambridge, UK; Loke Centre for Trophoblast Research, University of Cambridge, Downing Street, Cambridge CB2 3EG, UK; Cambridge Strategic Research Initiative in Reproduction, University of Cambridge, Cambridge, UK; British Heart Foundation, Cambridge Cardiovascular Centre for Research Excellence, University of Cambridge, Cambridge, UK

**Keywords:** Foetus, Cardiovascular, Sympathetic, Oxidative stress, Mitochondria, Metabolism, Epigenetics, Pregnancy, Exercise, Antioxidants

## Abstract

Pregnancy complicated by maternal obesity contributes to an increased cardiovascular risk in offspring, which is increasingly concerning as the rates of obesity and cardiovascular disease are higher than ever before and still growing. There has been much research in humans and preclinical animal models to understand the impact of maternal obesity on offspring health. This review summarizes what is known about the offspring cardiovascular phenotype, describing a mechanistic role for oxidative stress, metabolic inflexibility, and mitochondrial dysfunction in mediating these impairments. It also discusses the impact of secondary postnatal insults, which may reveal latent cardiovascular deficits that originated *in utero*. Finally, current interventional efforts and gaps of knowledge to limit the developmental origins of cardiovascular dysfunction in offspring of obese pregnancy are highlighted.

## Graphical Abstract

**Graphical Abstract:**
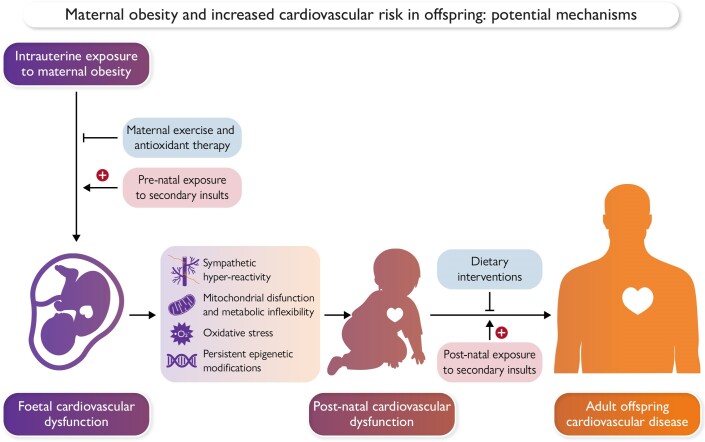
Intrauterine exposure to maternal obesity leads to offspring cardiovascular dysfunction through mechanisms including sympathetic hyper-reactivity, metabolic inflexibility, and mitochondrial dysfunction, as well as oxidative stress. Cardiovascular dysfunction originates in foetal life and persists into postnatal life through persistent epigenetic modifications. Interventions and secondary challenges may occur pre- and post-natally, which may impact progression to overt cardiovascular disease in adulthood. Created with BioRender.com.

**Key points**

- Exposure to maternal obesity during pregnancy results in increased risk of cardiovascular disease in offspring.
- Maternal obesity-induced cardiovascular dysfunction in offspring has origins *in utero*.
- Cardiovascular dysfunction in offspring of obese pregnancy arises and persists through sympathetic hyper-reactivity, mitochondrial dysfunction, metabolic inflexibility, and epigenetic modification via miRNAs.
- Exposure to secondary insults during adulthood, such as dietary modifications, stress, and ageing, can reveal latent cardiovascular impairments in offspring of obese pregnancy.
- Interventions established during gestation, such as maternal exercise or antioxidant supplementation, may be key in preventing cardiovascular dysfunction at its origin in offspring of obese pregnancy.

## Introduction

The World Health Organization describes obesity as a condition of epidemic proportions, with one in eight individuals and over 1.9 billion adults worldwide living with obesity.^1^ This rising prevalence of obesity matches a rising incidence of cardiovascular disease, which is now responsible for nearly 30% of all deaths in the UK.^2–6^ Increased adiposity leads to insulin resistance and hypertension, promoting a greater risk of cardio-metabolic disorders in obese individuals.^7,8^

The health implications of obesity rise exponentially to a much greater level of importance when considering maternal obesity.^9^ Over half of women in the UK are now overweight or obese during pregnancy.^10^ This is of the gravest concern as obesity during pregnancy not only has immediate detrimental effects on the mother but also on her children, thereby propagating adverse health conditions onto the next generation.^11^ Accumulating evidence derived from human studies and experimental animal models shows that maternal obesity can markedly increase the risk of cardiovascular disease in offspring,^6–30^ even when the progeny is fed a healthy diet and in the absence of them becoming obese.^23^ This highlights that it is something about the exposure of the embryo or foetus to an obesogenic environment during gestation itself that either triggers a foetal origin of cardiovascular dysfunction and/or increases susceptibility to heart disease in the adult offspring, consistent with the Developmental Origins of Health and Disease hypothesis.^31^

**Figure 1:**
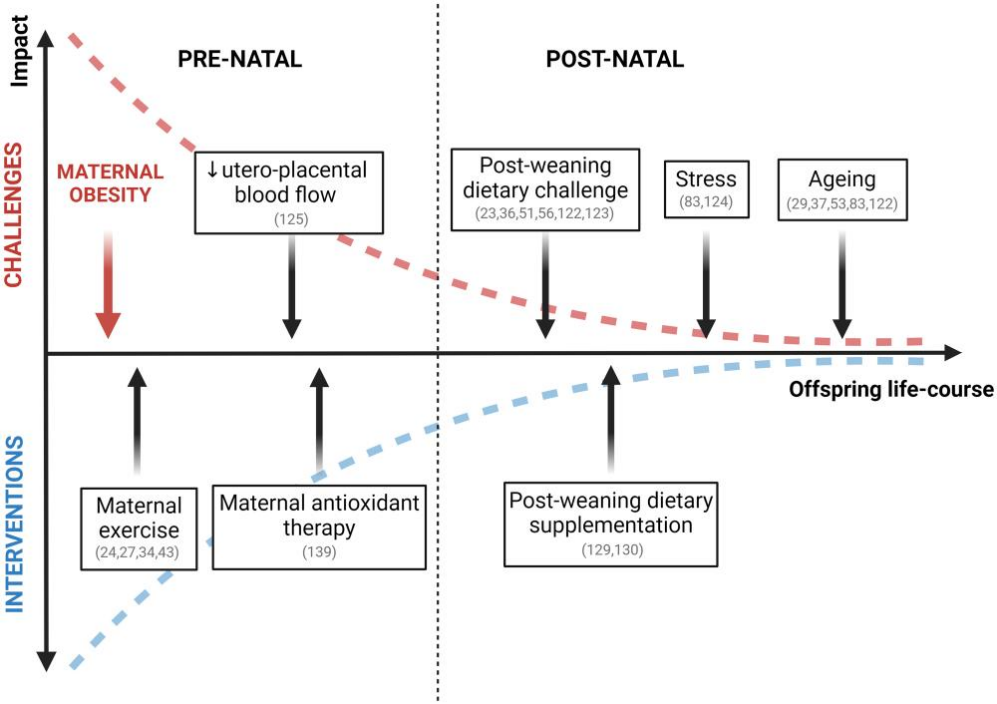
Timeline for intervention and secondary challenges over the life-course in offspring of obese pregnancy. The diagram shows that the younger we are the greater the impact that maternal obesity has upon us. Similarly, the opportunity for correction is greatest in younger life and diminishes progressively as we grow older. Therefore, candidate interventions should start as early as possible during the developmental trajectory, rather than waiting until disease is established. The diagram also shows that exposure to additional challenges in pre-natal and post-natal life, secondary to obese pregnancy, exacerbates the offspring cardiovascular dysfunction. The degree of impact of maternal obesity during pregnancy, superimposed challenges, and interventions is greatest in early pre-natal life, where the environmental sensitivity of progeny is highest, and falls exponentially across the offspring life-course. Key publications supporting statements are cross-referenced. Created with BioRender.com

In humans, the best evidence to support developmental origins of cardiovascular health and disease in offspring of obese pregnancy comes from studies in women who were obese during a first pregnancy, lost weight through bariatric surgery, and were leaner during a second pregnancy.^13,32,33^ These studies show that siblings born before bariatric surgery have signs of an increased cardiovascular risk compared with those born after surgery.^13,32,33^ Therefore, such studies highlight that a different environment in the same womb can programme a differential risk of heart disease in offspring of the same family. This provides compelling evidence in humans that the environment experienced during critical periods of development directly influences long-term cardiovascular health. Therefore, when considering strategies to reduce the burden of heart disease on every nation’s health and wealth, there needs to be a greater focus on prevention rather than treatment (*Figure 1*).

This review summarizes the evidence derived from human clinical studies and experimental animal models that reflects the impact of maternal obesity on the cardiac and vascular health of the adult offspring. Mechanistically, the work describes how alterations in the intrauterine environment during maternal obesity, such as foetal hypoxia and hyperinsulinaemia^34,35^ can lead to oxidative stress,^20,36^ mitochondrial dysfunction and metabolic inflexibility,^26,37–41^ contributing to sympathetic hyper-reactivity^21,22,36,42^ and cardiovascular dysfunction^21,37,38^ in offspring. How postnatal diet, stress, or ageing may reveal or exacerbate an underlying cardiovascular susceptibility originating *in utero* is also highlighted. Finally, the review focuses on current interventions, such as maternal exercise or dietary supplementation during pregnancy, against the developmental programming of cardiovascular dysfunction in offspring of obese pregnancy.

## Maternal obesity impacts offspring cardiovascular function during the postnatal period

### Evidence from human studies

**Figure 2:**
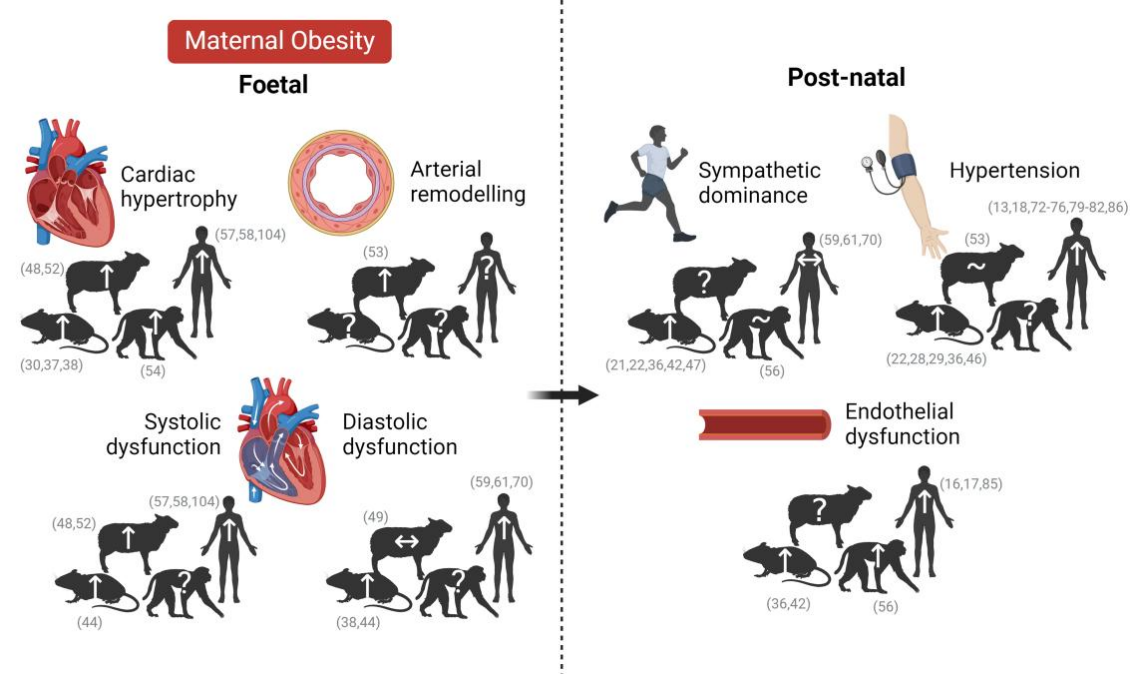
Cardiovascular phenotype of foetal and adult offspring of obese pregnancy in human and pre-clinical animal models. Maternal obesity induces cardiac hypertrophy,^30,37,38,48,52,54,57,58,104^ arterial remodelling,^53^ and systolic and diastolic dysfunction^38,104,44,48,49,52,57–59,61,70^ in the foetal offspring, leading to sympathetic dominance,^21,22,36,42,47,56,59,61,70^ endothelial dysfunction^16,17,36,42,56,85^, and hypertension^79–82,13,18,22,28,29,36,46,53,72–76,86^ in post-natal life. Key publications supporting statements are cross-referenced. Created with BioRender.com

An association between maternal obesity and offspring cardiovascular dysfunction postnatally is evident across many studies in humans (*Table 1* and *Figure 2*). Increased maternal body mass index (BMI) during pregnancy is associated with higher rates of hospital admissions due to cardiovascular events in adult offspring aged between 31 and 64^12,78^ and in a larger cohort aged between 27 and 76,^15^ with cardiovascular disease risk also higher in young human offspring aged between 1 and 25.^14^ Studies in children born to obese mothers reveal structural and functional cardiovascular alterations which likely drive this increased disease risk. In young children, increased maternal BMI during pregnancy is associated with left ventricular hypertrophy^16^ and greater epicardial adiposity.^17^ This is associated with diastolic dysfunction at 12 months of ages.^71^

**Table 1:** Cardiovascular outcomes in offspring of obese pregnancy

Species	Control diet	Experimental diet	Diet exposure period	Foetal/neonatal cardiovascular outcomes	Juvenile/adult offspring cardiovascular outcomes	References
Rodents						
Mouse *C57BL/6J*	3% fat, 7% sugar	16% fat, 33% sugar	6 weeks pre-pregnancy, throughout pregnancy and lactation	Altered cardiac lipidome (↑sphingomyelins and acyl-carnitines, ↓cholesteryl esters at d18.5) ↑ primary cardiomyocyte oleate oxidation and expression of genes associated with sterol, fatty acid and carnitine metabolism (at d18.5) ↑ cardiac HIF-1α and Ppara targets (d18.5)	↑ heart weight and cardiac hypertrophy (at 3 and 8 weeks) Re-expression of foetal genes (↑ *NPPB, ACTA1*, *MYH7:MYH6* ratio at 3 weeks) Cardiac systolic and diastolic dysfunction (at 12 weeks) ↓ LV ejection fraction and cardiac output (at 8 weeks) ↑ cardiac insulin and proliferative signalling (at 8 weeks) Cardiac oxidative stress (at 8 weeks) Cardiac and resistance artery sympathetic dominance (at 12 weeks) ↓ cardiac SERCA2a, total and phosphorylated troponin-I (at 12 weeks) ↑ active SBP and MAP but ↓ active heart rate and locomotion (at 12 weeks) Endothelial dysfunction in resistance arteries (at 12 weeks)	^20,21,23,24,39,42^
Mouse *C57BL/6J*	10% fat	40% fat, 20% sucrose soln.	4–6 weeks pre-pregnancy, throughout pregnancy and lactation	↑ heart weight (at d18.5) Altered cardiac expression of genes involved in metabolism (↑*Pparg*, *Cd36* and *Prkaa1*) with predicted ↓ neoplasia and DNA repair/synthesis and ↑ lipid synthesis and metabolism in males, ↓ immune cells and ↑ uptake of mono- and polysaccharides in females	Cardiac diastolic dysfunction in males (at 3, 6 and 24 months) and females (at 6, 9 and 24 months) ↑ heart weight in females (at 6 months) and ↑ cardiomyocyte cross-sectional area in males (at 3 months) ↑ cardiac foetal gene expression (at 3 months) ↑ cardiac Akt and mTOR signalling in males and ↓ERK1/2 signalling (at 3 months) Dysregulation of metabolism-related genes with ↑ expression of *Pparg* and targets related to lipid synthesis, storage and oxidation (at 6 months) ↑ myocardial mitochondrial fatty acid oxidation in males (at 6 months) ↓ myocardial glucose uptake in females (at 6 months)	^25,37^
Mouse *C57BL/6J*	10% fat	60% fat/45% fat	8 weeks pre-pregnancy, 45% fat throughout pregnancy	↓ placental vascular density		^43^
Mouse *C57BL/6J*	10% fat	60% fat	12 weeks pre-pregnancy, to necroscopy	Systolic dysfunction (↓ ejection fraction and fractional shortening) and diastolic dysfunction (↑ Tei index) at d16.5 ↑ cardiac ROS content (at d16.5)		^44^
Mouse *C57BL/6J*	25% fat, 3% sugar	60% fat, 13% sugar	6 weeks pre-pregnancy, throughout pregnancy and lactation		↑ absolute and relative left ventricular mass and internal diameter (at 8 weeks) ↓ fractional shortening in females (at 8 weeks) Circular cardiac mitochondrial morphology and disorganized sarcomere alignment ↓ cardiac mitochondrial oxygen consumption, CI and CII activity (at 8 weeks)	^26^
Mouse *C57BL/6N*	10% fat	45% fat	13 weeks pre-pregnancy, throughout pregnancy and lactation		Sympathetic dominance (↑ aortic adrenergic vasoconstriction at 14 weeks)	^27^
Mouse *C57BL/6*	10% fat	45% fat	6 weeks pre-pregnancy, throughout pregnancy and lactation		↑ systolic blood pressure (at 10 months)	^28^
Mouse *C57BL/6*	5% fat	60% fat	4 weeks pre-pregnancy, throughout pregnancy and lactation		↑ systolic and diastolic blood pressure (at 12 months)	^29^
Mouse *C57BL/6J*	21%	45%	4 weeks pre-pregnancy, throughout pregnancy and lactation		↑ systolic blood pressure (at 30 weeks) ↓ Acetylcholine-mediated vasorelaxation in femoral arteries (at 15 and 30 weeks) ↓ Basal NO production (at 30 weeks) ↑ oxidative stress in femoral arteries (at 15 weeks)	^36^
Rat *Sprague-Dawley*	4.3% fat	24% fat	From weaning, throughout pregnancy	↑ neonatal heart weight, cardiac fat deposition and apoptosis	↑ heart weight, cardiac fat deposition and apoptosis (at 1 and 3 months)	^30^
Rat *Sprague-Dawley*	18% fat	40% fat	4 weeks pre-pregnancy, throughout pregnancy and lactation	↑ heart:body weight ratio and myocardial lipid deposits (in neonates) ↓ heart rate and E:A ventricular filling ratio with ↑ isovolumetric contraction time (in neonates) ↓ cardiomyocyte basal oxygen consumption ↑ smaller, wider and fragmented cardiac mitochondria with ↓ mitochondrial fusion and fission and sex-specific alterations in expression of dynamism-related proteins (in neonates) Cardiac lipid peroxidation (in neonates) ↓ Cardiac FGF-activated PI3K/AKT signalling and ↑ PGC1α mitochondrial biogenesis signalling (in neonates)		^38,40,45^
Rat *Sprague-Dawley*	3% fat, 7% sugar	16% fat, 33% sugar	6 weeks pre-pregnancy, throughout pregnancy and lactation		↑ night-time MAP and MAP response to stress (at 4 and 12 weeks) ↓ HR in females (at 4 weeks) and males (at 12 weeks) Sympathetic dominance with ↑ renal tissue noradrenaline (at 4 and 12 weeks) ↑ changes in MAP to NO donor, α−adrenergic agonist and leptin, with ↓ baroreflex sensitivity of HR (at 12 weeks and 6 months)	^22^
Rat *Sprague-Dawley*	10% fat	60% fat	8 weeks pre-pregnancy, throughout pregnancy and lactation		↑ systolic and diastolic blood pressure (at 6 months) Altered renin-angiotensin pathway in adipose (at Day 1 and 6 months)	^46^
Rat *Sprague-Dawley*	4.5% fat	12.1% fat	12 weeks pre-pregnancy, throughout pregnancy and lactation		Mesenteric artery hypertrophy (at 4 months) Sex-specific alterations in vasodilator pathway dependence, with DNA methylation changes in vascular function genes (potassium channels and NO synthase in males, guanylate cyclase and angiotensin receptor I in females) (at 4 months)	^47^
Sheep						
Sheep *Rambouillet/Columbia* cross	100% NRC	150% NRC	60 days pre-pregnancy, to necroscopy at d75 or d135 (.5 or .9 gestation) or term	↑ left ventricular weight and left ventricular wall thickness (at d135) ↑ hypertrophic signalling (Foxo3a, mTOR and calcineurin pathways) and cardiac hyperplasia (at d75) ↑ cardiomyocyte cross-sectional area Irregular myofibre orientation, and perivascular fibrosis (at d75) ↓ cardiac contractile function in response to a high workload stress challenge (at d135) ↓ cardiomyocyte contractility, disrupted Ca^2+^ handling and ↑ myosin heavy chain β:α (slow twitch) expression (at d135) ↔ heart rate (at d135) ↓ cardiac insulin signalling Activation of fibrogenic genes (at d75 and d135), associated with increased collagen concentration (at d135) ↓ cotyledonary vascularity (at d75) ↑ aortic wall thickness and collagen:elastin ratio (at d135)	↑ Left and right ventricular wall thickness, ↑ myocardial collagen content and crosslinking and ↑ expression of pro-inflammatory cytokines (at 22 months, after a 3 month *ad libitum* feeding challenge) ↑ systolic blood pressure and heart rate at 2.5 months but ↓ systolic and diastolic blood pressure at 9 years Systolic dysfunction with ↓ fractional shortening, cardiac output and ejection fraction (at 9 years)	^48–53^
Non-human primates						
Baboon	12% fat, .61% sugar	45% fat, 12.58% sugar	4 months pre-pregnancy and throughout pregnancy to d165 (.9 gestation)	↑ myocardial fibrosis (d165) Dysregulated expression of cardiac microRNAs associated with cardiovascular disease ↑ cardiomyocyte proliferation rates		^54^
Japanese macaque	14% fat	32% fat	4+ years pre-pregnancy to necroscopy at d130 (.75 gestation)	↓ foeto-placental volume blood flow (at d120)		^55^
Japanese macaque	14% fat	36% fat	4+ years pre-pregnancy, throughout pregnancy and lactation		Altered aortic endothelial function depending on post-weaning diet: ↑ sensitivity to acetylcholine in control diet fed offspring, ↓ sensitivity and max relaxation to acetylcholine and in high fat diet fed offspring associated with hyperinsulinaemia (at 13 months, juvenile) ↑ aortic intima thickness and expression of pro-inflammatory markers and ↓ fibrinolytic signalling, regardless of post-weaning diet (at 13 months)	^56^

Humans				
Maternal phenotype	Offspring age	Foetal/neonatal offspring	Child/adult offspring	References
Obese: mean pre-pregnancy BMI 35 kg/m^2^ Control: mean pre-pregnancy BMI 21 kg/m^2^	Foetal: 14, 20 and 32 weeks of gestation	↑ interventricular septum thickness ↓ left and right ventricular global strain rate and strain (at 14, 20 and 32 weeks) ↓ tissue Doppler systolic and late diastolic velocities (at 20 and 32 weeks) ↔ heart rate		^57^
Obese: BMI >30 kg/m^2^ Control: BMI <30 kg/m^2^	Foetal: 25 weeks of gestation	↓ left ventricular ejection fraction and strain ↔ intra-ventricular septal thickness, myocardial performance index and mitral *E*/*A* ratio		^58^
Obese: pre-pregnancy BMI >30 kg/m^2^ Control: pre-pregnancy BMI 18.5–24.9 kg/m^2^	Foetal: 26–38 weeks of gestation	↑ heart rate and heart rate variability ↓ sympathetic dominance (LF:HF ratio)		^59^
Obese: pre-pregnancy BMI of ≥30 kg/m^2^ Control: pre-pregnancy BMI <25 kg/m^2^	Foetal: 26–38 weeks of gestation	Diastolic dysfunction with ↑isovolumetric relaxation time		^60^
Pre-pregnancy BMI range: 18.2–34.9 kg/m^2^	Foetal: during parturition	↑ sympathetic dominance (LF:HF ratio) with ↑ maternal BMI		^61^
Obese: pre-pregnancy BMI ≥30 kg/m^2^ Control: pre-pregnancy BMI 18.5–24.9 kg/m^2^	Foetal	ER stress and reduced NO synthesis in HUVECs ↓ NO-dependent dilation of umbilical vein segments in response to insulin ↓ Insulin signalling (↑ inhibitor phosphorylation of IRS-1 and ↓ activator phosphorylation of Akt)		^62,63^
Obese: BMI ≥25 kg/m^2^ Control: BMI <25 kg/m^2^	Foetal	Alterations in expression of genes related to lipid and mitochondrial metabolism in HUVECs		^64^
Obese: 32 weeks BMI ≥30 kg/m^2^ Control: 32 weeks BMI 18.5–24.9 kg/m^2^/37 weeks BMI <30 kg/m^2^	Foetal: 32 and 37 weeks of gestation	↑ umbilical artery pulsatility at 32 weeks ↔ umbilical artery pulsatility and foetal middle cerebral artery pulsatility at 37 weeks		^65,66^
Obese: parturition BMI ≥30 kg/m^2^ Control: parturition BMI 19.8–25.9 kg/m^2^	Foetal	↑ umbilical artery contractility		^67^
Obese: BMI ≥30 kg/m^2^ Control: BMI 18.5–24.9 kg/m^2^	Foetal	↓ chorionic plate artery endothelium-independent vasodilatation to nitric oxide donor		^68^
Obese (LGA infant): BMI 31.5 ± 2.5 kg/m^2^ Control (AGA infant): BMI 22.0 ± .6 kg/m^2^	Foetal	↓ chorionic plate artery endothelium-dependent vasodilatation to adiponectin in LGA pregnancies		^69^
Obese: Pre-pregnancy BMI ≥30 kg/m^2^ Control: pre-pregnancy BMI 10–15 kg/m^2^	Neonatal	↑ heart rate and ↓ heart rate variability ↓ left ventricular end diastolic volume and stroke volume		^70^
Overweight/obese: BMI ≥25 kg/m^2^ Control: < 25 kg/m^2^	Neonatal and 12 months.	↑ left ventricular posterior wall ↑end-diastolic and stroke volume		^71^
Obese: BMI >30 kg/m^2^ Control: BMI 18.5–24.9 kg/m^2^	1–25 years		↑ rates of cardiovascular disease	^14^
Obese: pre-pregnancy BMI ≥30 kg/m^2^ Control: pre-pregnancy BMI 18.5–24.9 kg/m^2^	4–6 years		↑ systolic and diastolic blood pressure ↔ autonomic balance	^18,72^
Obese: BMI >30 kg/m^2^ Control: BMI 18.5–24.9 kg/m^2^	6 years		↑ systolic blood pressure ↑ left ventricular eccentric hypertrophy and aortic root diameter with ↑ maternal BMI ↔ fractional shortening, *E*/*A* ratio, systolic and diastolic strain	^16,19,73,74^
Obese: gestational BMI of ≥30 kg/m^2^, body weight >91 kg or above normal by 110%–120% Control: mean gestational BMI 18 kg/m^2^	7–9 years		↑ epicardial adipose tissue thickness Arterial hypertrophy (↑ carotid intima-media thickness Reduced arterial compliance with ↓ arterial distensibility and strain and ↑ arterial stiffness	^17^
Obese: BMI >30 kg/m^2^ Control: BMI 18.5–24.9kg/m^2^	6–10 years		↑ systolic and diastolic blood pressure	^75,76^
Pre-bariatric surgery (obese): mean BMI 45 kg/m^2^ Post-bariatric surgery: mean BMI 27.6 kg/m^2^	9–15 years		↑ blood pressure in offspring born pre-bariatric surgery	^13^
Overweight/obese: BMI >25 kg/m^2^ Control: BMI 18.5–24.9 kg/m^2^	9–15 years		Maternal BMI positively associated with offspring waist circumference, and negatively associated with offspring cardiorespiratory fitness No association between maternal BMI and offspring blood pressure	^77^
Obese: BMI >30 kg/m^2^ Control: BMI 18.5–24.9 kg/m^2^	Adult: 27–76 years		↑ risk of hospital admission for cardiovascular event ↑ systolic and diastolic blood pressure	^12,15,78–80^

There is extensive evidence of cardiovascular dysfunction in offspring of obese pregnancy, in both pre-natal and post-natal life, across pre-clinical rodent, sheep and non-human primate models, and studies in humans. Diet % in kcal.

Abbreviations: AGA, average for gestational age (foetal weight); Akt, protein kinase B; *ACTA1*, actin alpha 1; BMI, body mass index; *Cd36*, a plasma membrane fatty acid translocase; *E*/*A* ratio, early/atrial ratio; FGF, fibroblast growth factor; HIF-1α, hypoxia-inducible factor 1 alpha; HUVEC, human umbilical vein endothelial cell; IRS-1, insulin receptor substrate 1; LF:HF ratio, low frequency: high frequency ratio; LGA, large for gestational age (foetal weight); MAP, mean arterial pressure; mTOR, mammalian target of rapamycin; *MYH6/7*, myosin heavy chain beta 6/7; NO, nitric oxide; *NPPB*, natriuretic peptide B; *Pparg/a*, nuclear peroxisome proliferator activated receptor.

Vascular alterations are also present, with increased aortic root diameter, arterial hypertrophy, and reduced arterial compliance in children of obese pregnancy.^16,17,85^ Together, these changes likely contribute to the increased prevalence of hypertension in children and adolescents born to mothers with obesity,^13,18,72–76,81,86^ showing a positive association between maternal pre-pregnancy BMI and offspring blood pressure even in the first year of life,^82^ that persists into adulthood.^79,80^ Evidence from human studies also indicates that cardiovascular disease risk in adult offspring is related to the degree of maternal obesity, with increased rates of cardiovascular disease only seen in offspring of mothers with obesity Grade II or higher (BMI over 35 kg/m^2^), which may be partly due to increased risk of additional complications such as neonatal asphyxia.^14^

### Evidence from animal studies

To further understand the cardiovascular phenotype of offspring of obese pregnancy, rodent, ovine, and non-human primate models of maternal obesity have been generated, each showing different technical and translational advantages and limitations, summarized in *Table 2*. Across mammalian preclinical models, exposure to maternal obesity during pregnancy leads to alterations in the heart structure and function in the progeny (*Table 1* and *Figure 2*). Rodent offspring of obese pregnancy show increased heart weight,^20,21,25,30,83,84^ with increased cardiomyocyte size.^20,21,25,26,83,84^ These alterations occur with the activation of hypertrophic signalling pathways including the re-expression of foetal genes^20,25^ and increased insulin signalling through AKT, ERK, and the mammalian target rapamycin (mTOR).^20,25^ Cardiac hypertrophy with greater myocardial collagen content has also been reported in adult offspring in an ovine model of maternal overnutrition.^51^

**Table 2:** Advantages and limitations of rodent, sheep and non-human primate models

	Rodents	Sheep	Non-human primates
Advantages			
Translational	Invasive, haemochorial placentation with trophoblast-mediated spiral artery remodelling, comparable to humans^87^ Feeding of highly translational ‘cafeteria’ and ‘Western-style’ diets to rodents leads to obesity and metabolic profiles comparable to humans^88^	Sheep are a precocial species with comparable cardiovascular developmental milestones to human pregnancy^89,90^ Certain breeds (e.g. welsh Mountain and Merino) are primarily uniparous with neonatal lamb weights comparable to full-term human infants^89^ Human and sheep placentas have a villous tree structure, concurrent exchange, and similar glucose and amino acid transport systems^91,92^ Treatments developed in sheep have been highly successful in humans e.g. antenatal maternal corticosteroid therapy for pre-term infants^93^	Relevant for highly species-dependent processes such as the mechanisms promoting parturition^94^ Similar hormonal changes and duration of menstrual cycle^95^
Technical	Short gestational length and reproductive cycle facilitates adult offspring and multi-generational studies Multiple pregnancy allows for study of sex differences within litter and using siblings for different outcomes (e.g. freezing vs. fixing) Cross-fostering and embryo transfer possible to isolate the critical periods of peri-conception, gestation and lactation	Chronic surgical instrumentation of the foetus is possible^89,96–99^ Longitudinal maternal and foetal blood sampling possible across gestation^89,96–99^ *Ex vivo* studies of foetal resistance arteries is possible^100^	Longitudinal maternal blood sampling possible across gestation *Ex vivo* studies on foetal cardiovascular system possible Life span and time to maturity are shorter than in humans
Limitations			
Translational	Rodents are an altricial species with cardiovascular developmental milestones that differ from humans^89,91^ Differences in basal metabolic rate and glucose disposal between rodents and humans may influence metabolic adaptations occurring with obesity and pregnancy Multiple pregnancy leads to differences from humans in foetal nutrient allocation	Lack of translational relevance for highly species-dependent processes such as the mechanisms promoting parturition^101^ Placentation is cotyledonary and synepitheliochorial^102^ Ruminant metabolism in sheep may result in different metabolic profile occurring with diet-induced obesity	Non-human primates show differences in placentation, with superficial blastocyst implantation, fewer interstitial trophoblasts and earlier onset of placental circulation^103^
Technical	Foetal long-term surgical instrumentation not feasible	Length of gestation and life span make adult offspring and multi-generational studies costly and time-consuming	Foetal instrumentation very limited Foetal blood sampling across gestation limited Length of gestation and life span make adult offspring studies costly and time-consuming

The primary pre-clinical models of maternal obesity in pregnancy are rodent, ovine and non-human primate models. Each of these models presents its own translational and technical advantages and limitations, which relate to the use of this animal model as a study of obesity and as a model of reproductive biology.

Maternal obesity also leads to systolic dysfunction in the adult offspring, with impaired cardiac output seen in mice^23,26,83^ and sheep.^53^ Reductions in left ventricular developed pressure,^21^ fractional shortening,^24,26,83^ ejection fraction,^23,24,26,83^ and heart rate^22,42^ all contribute to a lower cardiac output in rodent offspring. However, there are several discrepancies in heart rate and blood pressure changes in offspring of obese pregnancy; juvenile sheep show a trend towards tachycardia at 2.5 months associated with hypertension, both of which are absent at 9 years.^28^ In contrast, mouse offspring showing bradycardia at 1 and 3 months that reverses to tachycardia at 6 months, while showing a hypertensive phenotype at all time points.^16,35^ These differences may be age-dependent and influenced by a range of factors including species differences. Systolic dysfunction is associated with impairments in cardiomyocyte Ca^2+^ handling and activation of contractile proteins in mouse offspring.^21^ Diastolic dysfunction in mouse offspring of obese pregnancy results from an increase in left ventricular end-diastolic pressure,^21,37^ a reduced ratio of early-to-late left ventricular wall displacement and mitral inflow,^25,37,83^ together with longer isovolumetric relaxation time.^83^

Vascular alterations have also been reported, with mesenteric artery hypertrophy in adult rat offspring^47^ and thickening of the aortic intima in non-human primate offspring^56^ of obese pregnancy. Vascular dysfunction is evident, with a reduction in endothelium-dependent relaxation in resistance arteries of adult mice offspring of obese pregnancy.^36,42^ The effect on conduit arteries is less clear, with Macaque offspring of obese pregnancy showing increased aortic endothelial sensitivity to acetylcholine.^56^ In contrast, thoracic aorta endothelium-dependent and independent vasodilatation remained unaltered in adult mice offspring of obese pregnancy.^27^ This conflicting evidence may arise from species differences, study of resistance versus conduit vessels, and/or due to investigation of outcomes at different stages of maturity of the adult offspring. For instance, Macaque offspring were studied during the juvenile period^56^ while mouse offspring were studied as mature adults.^27^ Despite species and vascular bed differences, it is clear that offspring exposed to maternal obesity during gestation show alterations in vascular structure and function, which may contribute to the development of cardiovascular disease later in adulthood.

## Maternal obesity impacts offspring cardiovascular dysfunction during the prenatal period

While impacts on adult offspring cardiovascular risk are well established, there is now accumulating evidence suggesting that cardiovascular dysfunction in human offspring of obese pregnancy may originate before birth (*Figure 2*).

### Evidence from human studies

A reduction in bi-ventricular global strain is present in human foetuses of obese mothers at 14 weeks of gestation.^57,58,104^ Tissue Doppler imaging of foetal cardiac systolic and diastolic velocities and left ventricular ejection fraction reveals a reduction in all variables in human foetuses of obese mothers by 20–25 weeks, while an increased interventricular septum thickness becomes evident by 32 weeks of gestation.^57,58,104^ Basal foetal heart rate and heart rate variability are increased from mid-gestation in obese compared with healthy human pregnancies, associated with a reduction in the low frequency: high frequency (LF:HF) ratio.^59^ However, echocardiography studies during stimulated conditions, such as during parturition, revealed an increase in the foetal heart LF:HF ratio in obese pregnancy^61^ and neonatal recordings show decreased heart rate variability.^70^

Vascular dysfunction is also apparent in the human foetus of obese pregnancy, with increased umbilical artery constriction to serotonin,^67^ and impaired endothelium-dependent dilatation of the umbilical vein to insulin, an effect associated with vascular insulin resistance and oxidative and endoplasmic reticulum stress.^62,63^ These changes occur with an increase in the umbilical artery pulsatility index in offspring of obese women, measured at 32,^65^ but not at 37^66^ weeks of gestation. Impairments in endothelium-dependent and independent vasodilatation have also been reported in chorionic plate arteries of obese human pregnancy.^68,69^ However, no difference was found in the foetal middle cerebral artery pulsatility index with obese pregnancy.^66^

### Evidence from animal studies

Evidence derived from preclinical animal models, including rodent, sheep, and non-human primates, show cardiac structural alterations in offspring of obese pregnancy in prenatal life, which match the hypertrophy seen in adulthood (*Table 1* and *Figure 2*). Maternal obesity leads to increased heart weight in foetal mice^37^ and neonatal rats.^30,38^ The late-gestation foetal baboon shows increased cardiomyocyte proliferation and myocardial fibrosis in obese pregnancy, indicative of pathological hypertrophy.^54^ Similarly, foetal sheep exposed to maternal obesity show increased left ventricular weight and wall thickness with higher cardiomyocyte cross-sectional area, activation of hypertrophic signalling, and evidence of cardiac fibrosis.^48,52^

Evidence derived from preclinical animal models also supports impairments in cardiac function in foetal life during obese pregnancy. Foetal mice of obese pregnancy show systolic and diastolic dysfunction, with lower values for ejection fraction and fractional shortening, increased time spent in isovolumetric contraction and relaxation, and a reduction in the early atrial to ventricular (E:A) filling ratio.^44^ Foetal sheep cardiomyocyte contractility is also reduced in obese pregnancy, associated with impaired Ca^2+^ handling and an increased proportion of slow-twitch myosin heavy chains, resulting in impaired systolic function.^49,52^

Relatively few animal studies have explored the prenatal origin of vascular dysfunction in offspring of obese pregnancies, in part due to the practical size limitations of evaluating vascular reactivity of resistance circulations in foetal rodents. Placental vascular density is reduced during pregnancy in obese mice and sheep mothers,^43,105^ and foeto-placental blood flow is impaired in the obese Japanese macaques.^55^ Arterial hypertrophy is also evident, with an increased aortic wall thickness and in the aortic collagen:elastin ratio in the foetus of over-nourished ewes.^53^ Therefore, the available literature supports that obese pregnancy leads to alterations in the vascularization of tissues and in vascular structure in foetal life. However, the impact of maternal obesity on foetal vascular function appears entirely unknown, warranting further investigation.

## Mechanisms of cardiovascular dysfunction in offspring of obese pregnancy

**Figure 3:**
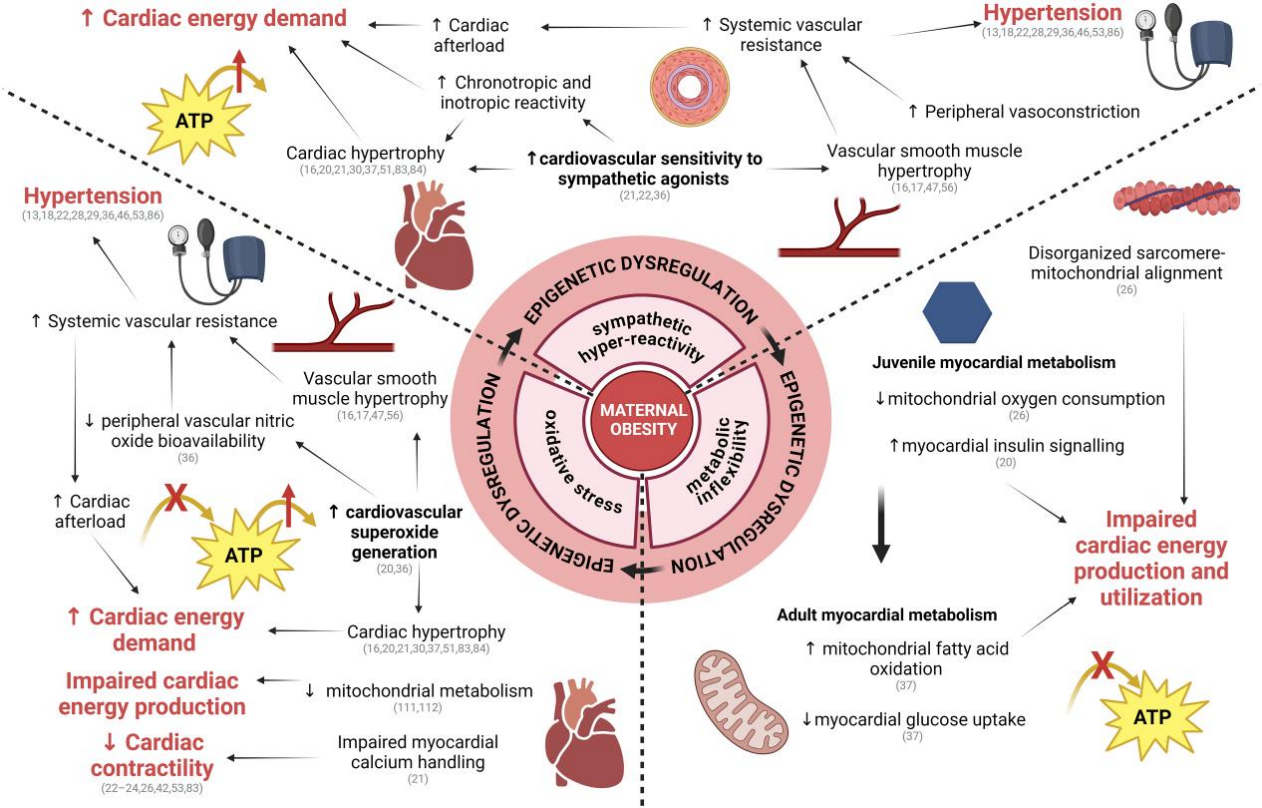
Mechanisms mediating cardiovascular dysfunction in offspring of obese pregnancy. Exposure to maternal obesity *in utero* leads to sympathetic hyper-reactivity,^21,22,36^ metabolic inflexibility^20,26,37^, and oxidative stress^20,36^ in the offspring, maintained through persistent epigenetic regulation,^52,120,122^ and eventually leading to overt cardiovascular dysfunction. Key publications supporting statements are cross-referenced. Created with BioRender.com

Animal models have been indispensable to identify causal mechanisms of cardiovascular disease programming by maternal obesity. Although several mechanisms have been proposed, the most prevalent leading to a persistent offspring phenotype can be summarized in four broad areas: sympathetic hyper-reactivity, mitochondrial dysfunction and metabolic inflexibility, oxidative stress, and epigenetic dysregulation including via miRNAs (*Figure 3*).

### Sympathetic hyper-reactivity

Sympathetic dominance in the cardiovascular system of adult offspring of obese pregnancy can be seen in many forms, including increased cardiac and vascular sensitivity to sympathetic agonists.^21,36,42^ There is also greater dose-dependent arterial pressure response to alpha-adrenergic agonists in adult offspring of obese rat pregnancy.^22^ Sympathetic hyper-reactivity of the peripheral vasculature can precipitate cardiovascular dysfunction as enhanced basal sympathetic tone and arterial hypertrophy independently promote an increase in peripheral vascular resistance, thereby increasing arterial blood pressure.^107^ Therefore, sympathetic hyper-reactivity contributes to the offspring hypertensive phenotype observed across several animal models of obese pregnancy.^22,28,29,36,46,53,86^ Increased arterial blood pressure also leads to a greater cardiac afterload, resulting in increased cardiac work. While an enhanced sympathetic drive helps to maintain cardiac output, it is known to be unsustainable, eventually becoming a hallmark of early-stage heart failure.^108,109^

An increased LF:HF ratio of foetal heart rate variability during parturition has been reported in pregnancies with increased maternal BMI in humans, consistent with a foetal origin of cardiac sympathetic dominance.^61^ However, a reduction in LF:HF has been measured during mid-to-late gestation,^59^ and no difference was found in the cardiac autonomic regulation of 5–6-year-old children born to obese compared with healthy weight mothers.^18^ This suggests that underlying sympathetic hyper-reactivity in the offspring heart resulting from maternal obesity may only be revealed in the presence of a superimposed challenge, such as during labour and delivery. However, detailed studies of the impacts of maternal obesity on foetal cardiovascular function during acute stressful conditions, such as during acute hypoxia, acute asphyxia, or acute hypotension, which trigger foetal sympathetic compensatory responses, await investigation.

### Mitochondrial dysfunction and metabolic inflexibility

Mouse offspring exposed to maternal obesity during gestation show increased cardiac insulin signalling^20^ and a reduction in mitochondrial oxygen consumption^26^ at 2 months of age. These data suggest that there may be increased dependence on glycolytic pathways for ATP generation. Cardiac mitochondria show circular morphology and a disorganized alignment relative to sarcomeres, which may result in poorer coupling of ATP production with consumption.^26^ However, -month-old mouse offspring of obese pregnancy show a reversed cardiac metabolic phenotype with increased mitochondrial fatty acid oxidation and a reduction in glucose uptake.^37^ This cardiac phenotype in adulthood may be an indication of metabolic inflexibility arising due to hyperinsulinaemia resulting from peripheral insulin resistance.^20^ Interestingly, a metabolic shift with increased dependence on fatty acid metabolism is characteristic of the cardiac phenotype in animal models of diabetic cardiomyopathy.^110^

The literature also supports that alterations in cardiac metabolism in offspring of obese pregnancy may originate in foetal life. Oleate oxidation is increased in foetal primary cardiomyocytes along with higher cardiac expression of lipid metabolism-related genes in foetal mice of obese pregnancy.^37,39^ Maternal obesity results in increased cardiac lipid deposition in neonatal rats, likely secondary to changes in myocardial lipid metabolism and hyperlipidaemia.^30,38^ Metabolic inflexibility is evident at this early stage, presenting a contrasting phenotype to adult offspring, with reductions in cardiac insulin signalling in the foetal sheep^49^ and fibroblast growth factor (FGF)-activated PI3K/Akt signalling in the neonatal rat^45^ during exposure to obese gestation. Mitochondrial fragmentation and reduced cardiomyocyte oxygen consumption in the neonatal rat also support that metabolic capacity is impaired and that this may be contributing to the reduced cardiac contractile function in offspring of obese pregnancy.^38,40^

### Oxidative stress

Mouse offspring of obese pregnancy show increased lipid peroxidation consistent with excess superoxide production, which limits basal and acetylcholine-induced nitric oxide production in the femoral artery.^36^ This shift in vascular oxidant tone results in endothelial dysfunction that becomes exacerbated over time, consistent with an increase in vascular oxidative stress in offspring of obese pregnancy.^36^ Cardiac oxidative stress is also evident in mouse offspring exposed to maternal obesity. Increased cardiac lipid peroxidation correlates with a reduction in the mitochondrial superoxide dismutase (MnSOD), and an upregulation of catalase levels.^20^ These alterations in expression of antioxidant enzymes are similar to those described in heart failure, and they are linked with impaired myocardial mitochondrial metabolism.^111,112^

Excess generation of reactive oxygen species (ROS) and increased HIF-1α target gene expression levels have both been reported in the hearts of foetal mice exposed to maternal obesity.^39,44^ As ROS generation is reported to stabilize HIF-1α, this is consistent with elevated oxidative stress.^39,44^ Neonatal rats of obese pregnancy also show higher levels of cardiac lipid peroxidation.^38^ Damage to mitochondrial metabolism due to oxidative stress further exacerbates existing perturbation of cardiac energy balance, with reduced flexibility of ATP production pathways and poorer coupling of myocardial ATP production and use. Therefore, oxidative stress creates and exacerbates impairments in systolic and diastolic dysfunction in offspring of obese pregnancy.

### Epigenetic regulation by miRNAs

Alterations in epigenetic signals, including DNA methylation, histone modifications, and miRNA expression, may provide a mechanism for persistent offspring cardiovascular dysfunction from foetal life into adulthood.^113,114^ Of particular interest are miRNAs, as their epigenetic dysregulation can modulate networks of genes in a coordinated fashion.^115^ MiRNAs are small non-coding RNAs that base-pair to specific sequences within the 3′ untranslated region of mRNA-target transcripts and act to decrease mRNA stability and/or block translation.^116^ Foetal cardiac miRNA expression is dysregulated by maternal high-fat feeding in non-human primates^54,106^ and in genetically obese-prone mice.^117^ Predicted targets of miRNAs dysregulated in the foetal baboon heart are p53, PPAR-γ, and HIF-1α, which are known to play key roles in cell cycle regulation, metabolism, and oxidative stress signalling, thus providing a mechanistic framework by which dysregulated miRNA expression could lead to increased risk of cardiovascular disease.^54,106^ MiRNA dysregulation has been shown to persist into adulthood, with miRNA-15b increased in the myocardium of adult mouse offspring and in the serum of human offspring exposed to obesity during pregnancy.^118^ MiRNA-15b is released in response to ischaemia-reperfusion of mouse hearts *ex vivo*, with increased release in hearts of offspring from obese pregnancy.^118^ MiRNA-15b overexpression reduces cardiomyocyte mitochondrial outer membrane stability and fatty acid oxidation *in vitro*, demonstrating a role of miRNA-15b in cardiac metabolism.^118^ Programmed changes in cardiac miRNAs are consistent with a growing body of evidence suggesting that miRNAs play an important role in the pathogenesis of cardiovascular disease (see^119^). The mechanisms by which an *in utero* obesogenic environment leads to permanent changes in miRNA expression are unknown but could involve programmed changes in DNA methylation and histone modifications of DNA regions regulating miRNA transcription. In addition to contributing to programming mechanisms, miRNAs could also be exploited as disease biomarkers^120^ and therapeutic targets.^121^

## Secondary insults reveal latent cardiovascular susceptibility in offspring of obese pregnancy

Offspring of obese pregnancy can show evidence of sympathetic hyper-reactivity, mitochondrial dysfunction, oxidative stress, and epigenetic dysregulation as the most prevalent or persistent phenotype, even decades after birth. However, overt cardiovascular dysfunction is not always seen. It is possible that such aspects of the cardiovascular phenotype in these offspring may enhance sensitivity to secondary insults that unveil latent susceptibility to future cardiovascular risk. Increasing evidence supports this concept, and focuses on alterations in diet, stress and ageing as likely secondary stressors (*Figure 1*).

### Post-weaning diet

Cardiac hypertrophy and inflammation are present in lambs exposed to maternal obesity during gestation only after a 12-week feeding challenge, compared with lambs of control pregnancy exposed to the same feeding challenge.^51^ While exposure to maternal obesity during gestation leads to cardiac hypertrophy and reduced ejection fraction in 8-week-old mouse offspring, the development of myocardial fibrosis and hypertension is only present at this stage in offspring exposed to an obesogenic post-weaning diet.^23^ Vascular dysfunction is also evident in macaque offspring of obese pregnancy dependent on post-weaning diet, with offspring on a control diet showing enhanced endothelium-dependent vasodilatation in the aorta, an effect which is reversed in offspring fed a high-fat diet.^56^ Importantly, in these pre-clinical studies, cardiovascular dysfunction is identified as compared with offspring of control pregnancy also exposed to the same altered post-weaning diet, demonstrating that maternal obesity leads to a heightened susceptibility to cardiovascular dysfunction induced by a dietary challenge.^23,51,56^ 15 week-old mouse offspring of obese pregnancy are hypertensive with impaired basal vascular nitric oxide production only when exposed to a high fat post-weaning diet.^36^ However, these changes were comparable to offspring of control pregnancy with high-fat post-weaning diet.^36^ An obesogenic post-weaning diet has also been shown to suppress the compensatory upregulation of myocardial fatty acid oxidation in offspring of obese pregnancy, and to increase expression of uncoupling proteins.^122^ Similarly, platelet hyperactivation is only observed in male mouse offspring of obese pregnancy which were also exposed to a high fat post-weaning diet.^123^ Therefore, a superimposed dietary challenge exacerbates cardiac dysfunction in adult offspring of obese pregnancy through structural, inflammatory, and metabolic pathways.

Possible mechanisms for increased sensitivity to a post-weaning dietary challenge in offspring of obese pregnancy include dysregulation of appetite control, poor nutrient handling, and metabolic inflexibility. Mouse offspring of obese pregnancy are hyperphagic,^42^ increasing susceptibility to diet-induced obesity. Mouse offspring of obese pregnancy also show increased serum insulin levels in the absence of hyperglycaemia, indicative of insulin resistance, resulting in greater metabolic vulnerability.^42^ For instance, mouse offspring of obese pregnancy show exacerbated hyperinsulinaemia following exposure to a high-fat/high-sugar post-weaning diet.^23^ Mouse offspring of obese pregnancy also show myocardial metabolic inflexibility, with increased dependence on fatty acid oxidation over glucose metabolism.^37^

Combined, hyperphagia and dysregulated glucose handling exacerbate disruption to the metabolic and endocrine *milieu* with a dietary challenge, imposing additional challenges to a heart that already has reduced flexibility in the metabolic pathways available for myocardial ATP production.

### Stress

Maternal obesity may also prime offspring to show dysregulated cardiovascular responses to stress, revealing a heightened vulnerability to cardiac injury. Mouse offspring of obese pregnancy show enhanced myocardial fibrosis, systolic and diastolic dysfunction compared with offspring of healthy pregnancy in response to a 2-week stress challenge.^83^ Similarly, mouse offspring from A^y^-mutant obese dams showed significantly higher infarct size than control offspring following an ischaemia-reperfusion challenge,^124^ indicating reduced coronary reserve to maintain cardiac function with the superimposed challenge. A plausible mechanism for enhanced sensitivity to stress is sympathetic hyper-reactivity. Rodent offspring of obese pregnancy show elevated cardiac and vascular sensitivity to adrenergic agonists, which may result in a greater increase in peripheral and coronary vascular resistance in the presence of stress, leading to increased cardiac afterload and poorer myocardial perfusion, alongside enhanced stimulation of cardiac hypertrophy.^21,36,42^

### Ageing

The onset of maternal obesity-induced hypertension is known to be age-dependent across a range of animal models (*Table 1*). Mouse offspring of obese pregnancy show no difference in systolic blood pressure at 4–6 months, but by 7–12 months show a significant elevation compared with age-matched controls.^29,122^ In contrast, juvenile sheep offspring exposed to maternal obesity during gestation show hypertension, which appears to resolve during adulthood.^53^ However, echocardiography reveals the progression of significant impairments in systolic function in ageing sheep offspring of obese pregnancy compared with ageing offspring of control pregnancy.^53^ Ageing in mouse offspring of obese pregnancy has also been associated with the development of both systolic and diastolic dysfunction.^37,83^ These preclinical studies highlight that it is the interaction between developmental exposure to maternal obesity and ageing which mediates cardiovascular dysfunction due to heightened susceptibility, not seen in aged offspring of control pregnancy.

While several studies highlight the impact of exposure to secondary insults postnatally, superimposed challenges in the prenatal environment may also play a significant role in exacerbating maternal obesity-induced cardiovascular dysfunction in offspring. For instance, a study in rats showed that uterine artery ligation in obese pregnancy results in increased relative heart weight and exacerbated alterations in arterial wall structure in 60-day-old offspring.^125^ Therefore, the interaction between maternal obesity and other intrauterine challenges may also be important in determining offspring cardiovascular health. However, our analysis highlights a gap in the literature of studies investigating the interaction between an *in utero* obesogenic environment and common stressors in foetal life, such as foetal hypoxia or excess foetal glucocorticoid exposure.

## Interventions against the developmental programming of cardiovascular dysfunction in offspring of obese pregnancy

Independent of whether secondary insults occur pre- or post-natally, their occurrence can reveal latent susceptibilities, leading to the expression of overt cardiovascular dysfunction in adult offspring of obese pregnancy later in life. This highlights the need for intervention, while also providing potential windows of opportunity for preventative therapy (*Figure 1*). To date, interventional strategies have focussed primarily on either maternal exercise or dietary supplementation during obese pregnancy.

### Maternal exercise

In mice, maternal exercise ameliorated maternal hyperinsulinaemia, prevented foetal hyperinsulinaemia, and normalized placental HIF-1α expression.^34^ These changes occurred with attenuation of cardiac hypertrophy and systolic dysfunction in 8-week-old adult offspring of obese pregnancy subjected to a maternal exercise intervention.^24^ Maternal exercise also alters the vasculature, improving placental vascularization in obese mouse pregnancy,^43^ and reversing vascular endothelial dysfunction in 23 week-old mouse offspring exposed to maternal obesity and a western diet post-weaning.^27^ Evidence from mouse models indicates that maternal exercise is an effective intervention to prevent cardiovascular disease programming, with protective effects observed in offspring even when using mild maternal exercise regimes that do not result in the normalization of maternal weight. Exercise interventions that improve the maternal metabolic phenotype, despite no effect on maternal BMI, may prevent the development of oxidative stress and metabolic inflexibility in the offspring cardiovascular system, leading to a reduced cardiovascular risk in offspring. This is an important message to convey to overweight women, that despite having no effect on their body weight, exercise during pregnancy still benefits the cardiometabolic health of their offspring.

Lifestyle interventions, such as maternal exercise, have been trialled in human subjects with no significant improvement in neonatal cardiac structure or function.^126^ However, a recent systematic review of randomized controlled trials highlighted that maternal lifestyle interventions, such as diet and physical activity, reduced cardiac remodelling and improved systolic and diastolic function in children exposed to maternal obesity in pregnancy.^127^ Interestingly, maternal lifestyle interventions did not have any effects on offspring blood pressure across trials,^127^ consistent with the persistence of offspring hypertension in a mouse model of maternal obesity with exercise intervention during pregnancy.^24^ However, with poor adherence to physical activity guidelines in pregnant women,^128^ alternative intervention strategies will likely need to be considered.

### Offspring and maternal dietary supplementation

Intervention through offspring dietary supplementation with glucose-lowering berberine has been shown to improve cardiac function, together with improved cardiac mitochondrial function in mouse offspring exposed to gestational diabetes.^129,130^ However, evidence points towards a foetal origin of cardiac dysfunction in obese pregnancy, and so prevention by maternal treatment during pregnancy compared with postnatal intervention may increase the effectiveness of the approach, providing optimal protection against offspring cardiovascular dysfunction (*Figure 1*). Several studies have reported that maternal antioxidant treatment is effective in protecting against cardiovascular dysfunction in offspring exposed to hypoxic pregnancy by attenuating oxidative stress in the placenta and the foetal cardiovascular system.^89,100,131–136^ Therefore, as offspring exposed to maternal obesity also show oxidative stress, maternal antioxidant therapy may provide an effective intervention against the programming of cardiovascular dysfunction in offspring of obese pregnancy. For example, antioxidant treatment of obese mice rescues oocyte mitochondrial dysfunction^137^ and oxidative stress.^138^ Treatment of obese mice with the antioxidant pyrroloquinoline quinone from conception and throughout lactation increased adult offspring oxidative defences and metabolic flexibility.^139^ Whether the beneficial effects of maternal antioxidant treatment during obese pregnancy extend to protection against the programming of cardiovascular dysfunction in offspring remains to be tested.

**Figure 4:**
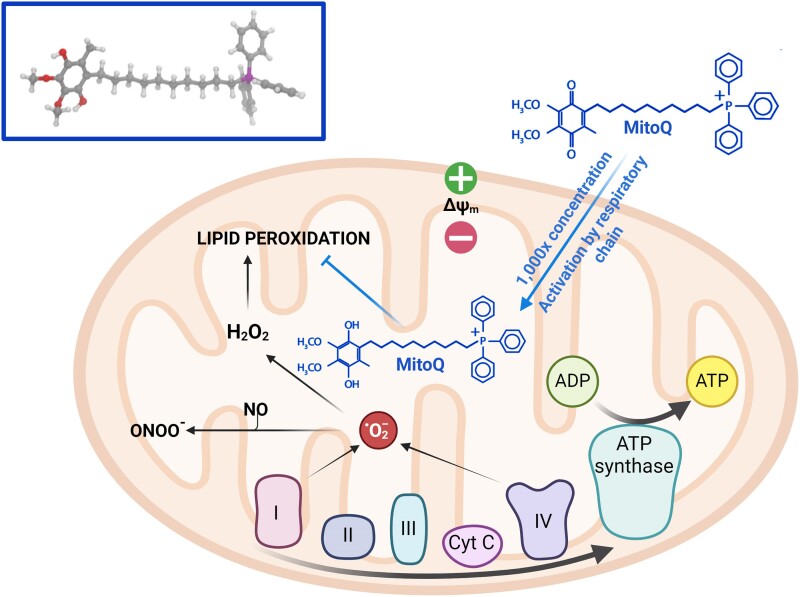
MitoQ: a mitochondria-targeted antioxidant. MitoQ is composed of a lipophilic triphenylphosphonium cation covalently attached to a ubiquinol antioxidant.^140–142^ The lipophilic cations facilitate free movement of MitoQ through phospholipid bilayers, while the triphenylphosphonium cation concentrates MitoQ ∼1000 fold within the mitochondria, driven by the large mitochondrial membrane potential.^140–142^ MitoQ is reduced by the respiratory chain to its active ubiquinol form once inside the mitochondrial matrix.^140–142^ This activated ubiquinol form of MitoQ inhibits lipid peroxidation, ameliorating mitochondrial damage.^140–142^ Created with BioRender.com

A key limitation of translating antioxidant therapies to human populations lies in identifying a safe, but effective dose. For example, the maternal supplementation with the antioxidant vitamin C during rat pregnancy has been shown to be protective against cardiovascular dysfunction of adult rat offspring exposed to chronic hypoxia *in utero*, however, the dose used was over 50 times the dose given to pregnant women in clinical trials.^133^ Therefore, there is an urgent need to identify alternative antioxidant therapies with increased human translational potential. Mitochondria are a major site of ROS production, therefore targeting these organelles should be one of the most effective antioxidant strategies. However, conventional antioxidants are ineffective because they cannot penetrate the mitochondria. A mitochondria-targeted ubiquinone that overcomes the problem of direct delivery to the mitochondria has now been developed (*Figure 4*). MitoQ is composed of a lipophilic triphenylphosphonium cation covalently attached to a ubiquinol antioxidant.^140–142^ Lipophilic cations can easily move through phospholipid bilayers without requiring a specific uptake mechanism. Therefore, the triphenylphosphonium cation concentrates MitoQ several hundred-fold within the mitochondria, driven by the large mitochondrial membrane potential.^140–142^ Only within the mitochondria, MitoQ is reduced by the respiratory chain to its active ubiquinol form, which is a particularly effective antioxidant that prevents lipid peroxidation and mitochondrial damage.^140–142^ The benefits of MitoQ have been revealed in a range of *in vivo* studies in rats and mice and have also been assessed in two Phase II human trials.^143–147^ In contrast to vitamin C and other conventional antioxidants, MitoQ demonstrates no pro-oxidant activity at high doses^143^ and long-term administration to mice^145^ and to human patients in Phase II trials, including one that lasted 12 months and revealed no toxicity.^146,147^ However, the antioxidant benefits of MitoQ in protecting the foetal and adult cardiovascular system in offspring of obese pregnancy remain to be investigated.

## Concluding remarks

There is extensive evidence derived from human studies and preclinical animal models for the programming of an increased risk of cardiovascular disease in offspring exposed to maternal obesity *in utero* (*Table 1* and *Figure 2*). Cardiovascular susceptibility in offspring has an early origin, with many aspects of the cardiac dysfunctional phenotype emerging in foetal life across mammalian species. This suggests that candidate interventions should start as early as possible during the developmental trajectory, rather than waiting until disease is established and has become irreversible. The effects of novel treatments like mitochondria-targeted antioxidant therapy during obese pregnancy in preclinical animal models should be explored. The literature also highlights a limited understanding of how vascular structure and function is altered in offspring of obese pregnancy before birth. Large mammalian animal models permitting functional assessment of foetal vascular reactivity in resistance circulations must be employed to address this gap in our knowledge. This review also addressed key programming mechanisms linking maternal obesity with offspring cardiovascular dysfunction, including sympathetic hyper-reactivity, the development of oxidative stress, mitochondrial dysfunction, metabolic inflexibility, and epigenetic dysregulation via miRNAs. Data also support that exposure to a secondary insult in adult life, or even the process of ageing, often reveals latent impairments in the cardiovascular system in offspring of obese pregnancy. It is likely that secondary insults occurring prenatally in offspring of obese pregnancy may also exacerbate latent susceptibility to cardiovascular dysfunction. Therefore, further research is required to understand how maternal obesity may impact the foetal cardiovascular defence to common acute stresses *in utero*, such as acute foetal hypoxia, acute foetal asphyxia, or acute foetal hypotension. In turn, further research is also required to understand how longer-term intrauterine complications in adverse pregnancy, such as chronic foetal hypoxia or excess foetal glucocorticoid exposure, may interact with maternal obesity to affect cardiovascular function in offspring.

## Supplementary data

Supplementary data are not available at *European Heart Journal* online.

## Declarations

### Data Availability

No data were generated or analysed for or in support of this paper.
